# Construction of ceRNA network to identify the lncRNA and mRNA related to non-small cell lung cancer

**DOI:** 10.1371/journal.pone.0259091

**Published:** 2021-10-29

**Authors:** Kui Xiao, Yang Wang, Lihua Zhou, Jufen Wang, Yaohui Wang, De Tong, Zhiruo Zhu, Jiehan Jiang

**Affiliations:** 1 Department of Respiratory and Critical Care Medicine, The Second Xiangya Hospital of Central South University, Changsha, Hunan, China; 2 Research Unit of Respiratory Disease, Central South University, Changsha, Hunan, China; 3 The Respiratory Disease Diagnosis and Treatment Center of Hunan Province, Changsha, Hunan, China; 4 Department of Pathology, The Second Xiangya Hospital of Central South University, Changsha, Hunan, China; 5 Department of Pulmonary and Critical Care Medicine, University of South China Affiliated Changsha Central Hospital, Changsha City, Hunan Province, China; University of Science and Technology Liaoning, CHINA

## Abstract

**Background:**

Non-small cell lung cancer (NSCLC) harms human health, but its pathogenesis remains unclear. We wish to provide more molecular therapeutic targets for NSCLC.

**Methods:**

The NSCLC tissue and normal tissue samples were screened for genetic comparison in the TCGA database. The predicted lncRNA and mRNA in BEAS2B and A549 cells were detected.

**Results:**

Volcano plot displayed differentially expressed lncRNAs and mRNAs in adjacent tissues and NSCLC tissues. The survival curve showed that the lncRNA and mRNA had a significant impact on the patient’s survival. The results of GO term enrichment analysis indicated that mRNA functions were enriched in cell cycle-related pathways. In the ceRNA interaction network, 13 lncRNAs and 20 miRNAs were found to have an interactive relationship. Finally, 3 significantly different lncRNAs (LINC00968, lnc-FAM92A-9 and lnc-PTGFR-1) and 6 mRNAs (CTCFL, KRT5, LY6D, TMEM, GBP6, and TMEM179) with potential therapeutic significance were screened out. And the cell experiment verified our results.

**Conclusion:**

We screened out clinically significant 3 lncRNAs and 6 mRNAs involved in the ceRNA network, which were the key to our future research on the treatment of NSCLC.

## 1 Introduction

Non-small cell lung cancer (NSCLC) is the major type of lung cancer, with an annual mortality rate of 1.6 million worldwide [1, 2]. Smoking was the main cause of NSCLC. However, the NSCLC rate of non-smokers was also increasing year by year due to environmental pollution [3, 4]. The current treatment for NSCLC was based primarily on traditional radiotherapy, chemotherapy and surgery. However, the prognosis of patients with these treatments was poor and low survival rate [5].

Long non-coding RNA (lncRNA), which existed in the cytoplasm and nucleus, was not involved in protein transcription and translation. But lncRNA participates in gene expression and signal transduction pathways [6, 7]. In recent years, 100,000 lncRNAs have been found in the human genome [8, 9]. LncRNAs used different potential mechanisms for carry out genetic regulation, chromatin remodeling, gene transcription, protein transport, cell differentiation, etc. [10, 11]. The role of lncRNA in regulating gene expression has aroused the interest of scientists in the research and treatment of various cancers. Besides, tumor cell proliferation, angiogenesis, cancer cell metastasis, and chemotherapy resistance were regulated by the tumor microenvironment (TME). The lncRNA secreted from exosomes participates in the signal transduction of tumor mechanism in TME [12] and impacts the growth, differentiation, and metastasis of tumor cells [13].

At present, some lncRNAs have been experimentally reported to be related to the development of NSCLC. For example, Guo et al. found lncRNA TUG1 was lowly expressed in non-small cell lung cancer (NSCLC) clinically [14]. Luo et al. found that in cell experiments, lncRNA TRERNA1 promoted the malignant progression of NSCLC by regulating FOXL1 [15]. Liu et al. inhibited the progression of NSCLC by knocking down lncRNA-XIST through the pyrolysis mediated by the miR-335/SOD2/ROS signaling pathway [16]. lncRNAs have been reported to regulate p53 (a tumor suppressor gene) through TME, such as the repressor lincRNA-p21 in the p53 pathway [17]. The p53 effector Loc285194 could inhibit the growth of tumor cells [18].

Various lncRNAs competed with limited miRNAs [19, 20] to form an endogenous ceRNA interaction network. The interacting genes could be identified by constructing the ceRNA network. For example, Rui-Sheng Zhou et al. found 10 mRNAs interact with 8 miRNAs in snake squamous cell carcinoma by constructing a ceRNA network [21]. Meanwhile, the construction of the ceRNA network helped predict the regulation mechanism of lncRNA-miRNA-mRNA on the disease to find potential targets for the treatment of the disease. Kong et al. found 33 lncRNA-miRNA-mRNA pathways that regulated NSCLC after constructing the ceRNA network of NSCLC, which provided a basis for subsequent research on NSCLC therapeutic targets [22].

Bioinformatics relies on "big data" algorithms, making it easier to identify novel drug targets and biomarkers [23]. It has been widely used for data integration before basic research. These specific processes include establishing large databases, drug therapy positioning prediction, evaluating the correlation between targets and diseases, and analyzing "big data" to identify bioinformatics algorithms for new drug targets or biomarkers [23–25]. Our study selected RNA sequences of NSCLC tissue samples and adjacent tissues from LNCipedia, TCGA, lncBase, and miRTarBase databases. Various biological information methods were performed to analyze and compare the above RNA sequences [26–28]. After finding the NSCLC relative lncRNA sequence and mRNA sequence, survival curves analysis and function prediction analysis were performed, respectively. The ceRNA network of NSCLC was utilized to screen out clinically significant lncRNA and mRNA. We hope this research could provide more molecular therapeutic targets for the treatment of NSCLC.

## 2 Methods

### 2.1. NSCLC expression data set

The Cancer Genome Atlas (TCGA) database (https://portal.gdc.cancer.gov/) is a platform dedicated to storing various types of tumor information. We selected 52 NSCLC tissue samples and 8 para-cancerous tissue samples from the TCGA database, compared and analyzed them.

### 2.2. Differentially expressed genes in lncRNA and mRNA data

We used Bioconductor’s R language DEseq2 package to analyze the differential expression of the sequences of the adjacent tissues and NSCLC tissue samples. The screening criteria were genes with |logFC|>2 and P-value<0.05 under the TCGA data set. Through single factor Cox regression analysis of all lncRNAs in the selected adjacent tissues and NSCLC tissue samples. volcano plot, box plot and cluster heat map were drawn for lncRNAs. Volcano plots were drawn for mRNAs.

### 2.3. Survival curve analysis

RTCGA NSCLC clinical data were used to screen mRNA. Kaplan-Meier survival curves of lncRNA and mRNA were drawn respectively (http://kmplot.com/analysis/). Based on the median value, expressions of genes were divided into high expression and low expression. The log-rank test was performed to screen out lncRNA and mRNA related to the prognosis of NSCLC. p<0.05 was significant.

### 2.4. GO term and KEGG pathway analysis

DEG was uploaded to DAVID (a database for annotation, visualization and integrated discovery) version 6.8 (http://david.ncifcrf.gov). Then we used the clusterProfiler package to analyze the GO term (http://www.bioconductor.org/packages/release/bioc/html/clusterProfiler.html) and KEGG pathway (http://www.genome.jp/kegg/). We detected the function of mRNA from three aspects: cell composition, molecular function and the biochemical process by GO term. KEGG pathway enrichment analysis was used to explain which pathways of the human body play a role in differential genes. P <0.05 was considered significant. After analyzing the gene set obtained by GO term and KEGG pathway analysis, we used gene enrichment analysis (GSEA) to analyze its expression changes. The javaGSEA desktop application was downloaded from http://software.broadinstitute.org/gsea/index.jsp, the multiple change values of all genes were exported. And the GSEA version 2.2.0 was used to analyze the GSEA. To calculate the enrichment score (ES) of a pathway, GSEA checked genes step by step from the top to the bottom of the ranking list. If the gene was part of the pathway, ES was increased. Otherwise, the score was decreased. According to GSEA analysis, we have obtained several pathways related to NSCLC.

### 2.5. Construction of ceRNA network

The lncBase database (www.microrna.gr/LncBase) was dedicated to recording the interaction between lncRNA and miRNA. And the miRTarBase database (http://miRTarBase.mbc.nctu.edu.tw/) was a database dedicated to collecting miRNA-mRNA targeting relationships (MTI, MicroRNA-Target Interactions) supported by experimental evidence. Before performing preliminary statistical analysis, we downloaded miRNA-mRNA interaction information from miRTarBase (http://mirtarbase.mbc.nctu.edu.tw/), and got lncRNA-miRNA interaction information from LncBase (http://carolina.imis.athena-innovation.gr/diana_tools/web/index.php?r=lncbasev2%2Findex-experimental). We entered each target lncRNA, miRNA, and mRNA in the data box. Then the detection was performed to obtain the relationship data between miRNA and lncRNA or mRNA. Cytoscape v.3.5.1 was used to select miRNA-regulated lncRNA and mRNA, showing significant results in hypergeometric tests and correlation analysis, to construct a ceRNA network.

### 2.6. Cell culture and treatment

Human bronchial epithelialcells (BEAS2B) and human NSCLC cells (A549) were obtained from Procell Life Science&Technology Co., Ltd. BEAS2B cells were maintained in LHC-9 medium supplemented with 1% penicillin and streptomycin. A549 cells were maintained in Dulbeccos modified Eagles medium (DMEM) supplemented with 10% fetal bovine serum (FBS; Invitrogen). Then, A549 cells were divided into three groups, Control, si-NC, and si-LINC00968. Briefly, Control group cells were cultured normally, LINC00968 negative control (si-NC) and si-LINC00968 small interfering RNA (si-LINC00968) were transfected into A549 cells. All cells were cultured at 37°C in a humidified incubator with 5% CO_2_.

### 2.7. Quantitative Real-time PCR (qRT-PCR)

The cells in good growth condition were collected. According to the instructions of the Trizol kit, 1 mL Trizol was added to the samples of each group. After mixing well, the samples were split in the chamber for 3 min to extract the total RNA. The first strand of cDNA was synthesized with miRNA cDNA Synthesis Kit. Next, according to the HiFiScript cDNA Synthesis Kit manufacturer’s instructions, the cDNA was reverse transcribed from the total tissue mRNA. Primer 5 software was used to design the primer sequence (Table 1). The abundance of mRNA was measured on qRT-PCR system by UltraSYBR Mixture. β-actin was utilized as the endogenous control gene. According to the comparison threshold method (2^-ΔΔCt^), the relative expression level was calculated.

**Table 1:** The primers used in this study.

Primer ID	5’-3’
hsa-miR-30a-3p-RT	CTTTCAGTCGGATGTTTGCAGC
hsa-miR-338-3p-RT	TCCAGCATCAGTGATTTT
hsa-miR-451a-RT	AAACCGTTACCATTACTGAGTT
hsa-miR-4732-3p-RT	GCCCTGACCTGTCCTGTTCTG
LINC00968-F	GGGTAACTTCAGGTGGAGCC
LINC00968-R	AGGAGCTCGCTTCTTTGGTT
CIBAR1-F	TCTACACTGCTGCCTACCAGAA
FAM92A-9-R	CTAGATCAGGGTTTGGCAAGTT
PTGFR-F	AATCCAGCTCCTGGCGATAA
PTGFR-R	ATGTTGCCTCTCCATCCACAC
CTCFL-F	AGTAAATTGAAGCGCCATGTCC
CTCFL -R	GTATTTTCATGGTCCCGCTCT
KRT5-F	GGCGAGGAATGCAGACTCA
KRT5-R	ACTGCTACCTCCGGCAAGA
LY6D -F	TCCAGCAACTGCAAGCATTC
LY6D -R	CTCCACTGTGTTCGTGGTCT
TMEM1-F	AGCTGAGCGACCAGAAACAG
TMEM1-R	AACGAGAGTCCCTCTTCATCT
GBP6-F	GGTGCTATAGAGCAGGGTGAC
GBP6-R	GGTGCTCATGCTGTTGTAGAC
TMEM179-F	GCCATGGCGCTCAACAATTT
TMEM179-R	CACCACGAAGCTGAACAGGA
Bax-F	TCACTGAAGCGACTGATGTCCC
Bax-R	ACTCCCGCCACAAAGATGGTC
BCL2-F	AGCTGCACCTGACGCCCTT
BCL2-R	ACATCTCCCGGTTGACGCTCT
PI3K-F	TGCGTCTACTAAAATGCATGG
PI3K-R	AACTGAAGGTTAATGGGTCA
MTOR-F	CCAAAGGCAACAAGCGATCCCGAA
MTOR-R	CTCCAAGTTCCACACCGTCCA
wnt3a-F	TGTTCCACTGGTGCTGCTAC
wnt3a-R	TTTAGGTGGGAGTCCTGCTC
β-actin-F	ACCCTGAAGTACCCCATCGAG
β-actin-R	AGCACAGCCTGGATAGCAAC

### 2.8. Western Blot (WB) assay

The cells in good growth condition were collected. After rinsing with PBS, RIPA lysate was used to break up cells. The protein concentration was determined according to the instructions of BCA protein quantitative kit. The same amount of protein was separated by 10% SDS-PAGE gel and then transferred to the PVDF membrane. The membranes were incubated with the primary antibody CTCFL (60079-1-Ig, 1: 1000, proteintech), KRT5 (66727-1-Ig, 1: 10000, proteintech), LY6D (17361-1-AP, 1: 300, proteintech), TMEM (20995-1-AP, 1: 500, proteintech), GBP6 (23001-1-AP, 1: 1000, proteintech), TMEM179 (24799-1-AP, 1: 600, proteintech), β-actin (60008-1-Ig, 1: 5000, proteintech) overnight at 4°C. Then the secondary antibody HRP goat anti-mouse IgG (SA00001-1, SA00001-1, proteintech) and HRP goat anti-rabbit IgG (SA00001-2, 1: 6000, proteintech) were used to incubate the membrane for 90 min at room temperature. ECL was performed to develop the color of the substrate.

### 2.9. Immunofluorescence (IF)

Each group of cells (5 × 10^4^) were plated on coverslips and cultured for 24 h. The cells were fixed with 4% paraformaldehyde for 30 minutes. After rinsing with PBS, all samples were covered with 100 μL Proteinase K working solution. The samples were incubated with Equilibration Buffer for 10–30 min at room temperature. Then, 50 μL TdT incubation buffer was added to Samples and incubated for 1 h at room temperature. The cells were counterstained with DAPI (Sigma-Aldrich) to observe the nucleus.

### 2.10. Cell apoptosis assay

The cells were collected and washed twice with PBS. 500 μL Binding Buffer were added into all groups of cells to suspend the cells. Annexin V-FITC was added into cells and mixed well. Then cells were added 5 μL Propidium Iodide at room temperature and reacted in the dark for 5–15 minutes; the cells were detected by flow cytometry.

### 2.11. Statistical analysis

Graphpad Prism 8.0 software (GraphPad Software, San Diego, California, USA) was used for statistical analysis. The student’s t-test was used to analyze the differences between the two groups of data. The data comparison between groups conforming to the normal distribution uses one-way ANOVA. And Tukey’s performs post-hoc test. P <0.05 indicates that the difference is statistically significant.

## 3 Results

### 3.1. The expression of lncRNA in NSCLC tissues and adjacent tissues

**Fig 1:**
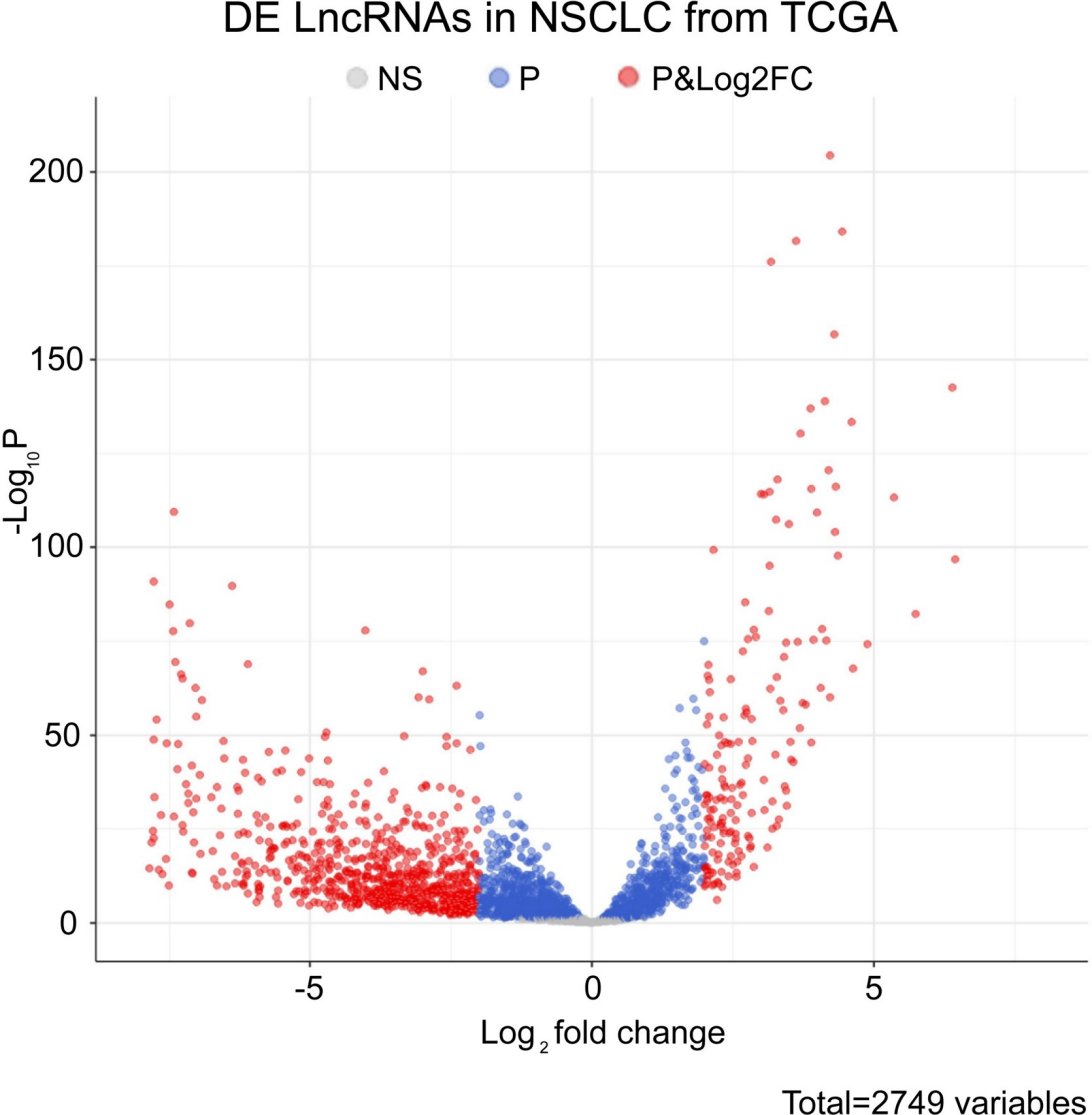
The differential expression of lncRNA volcano plot in cancer tissues and adjacent tissues. The ordinate is the p-value, and the log2 value of the screening condition was used as the abscissa. Upregulated genes and downregulated genes were marked in light red (|logFC|>2 and P-value<0.05).

**Fig 2:**
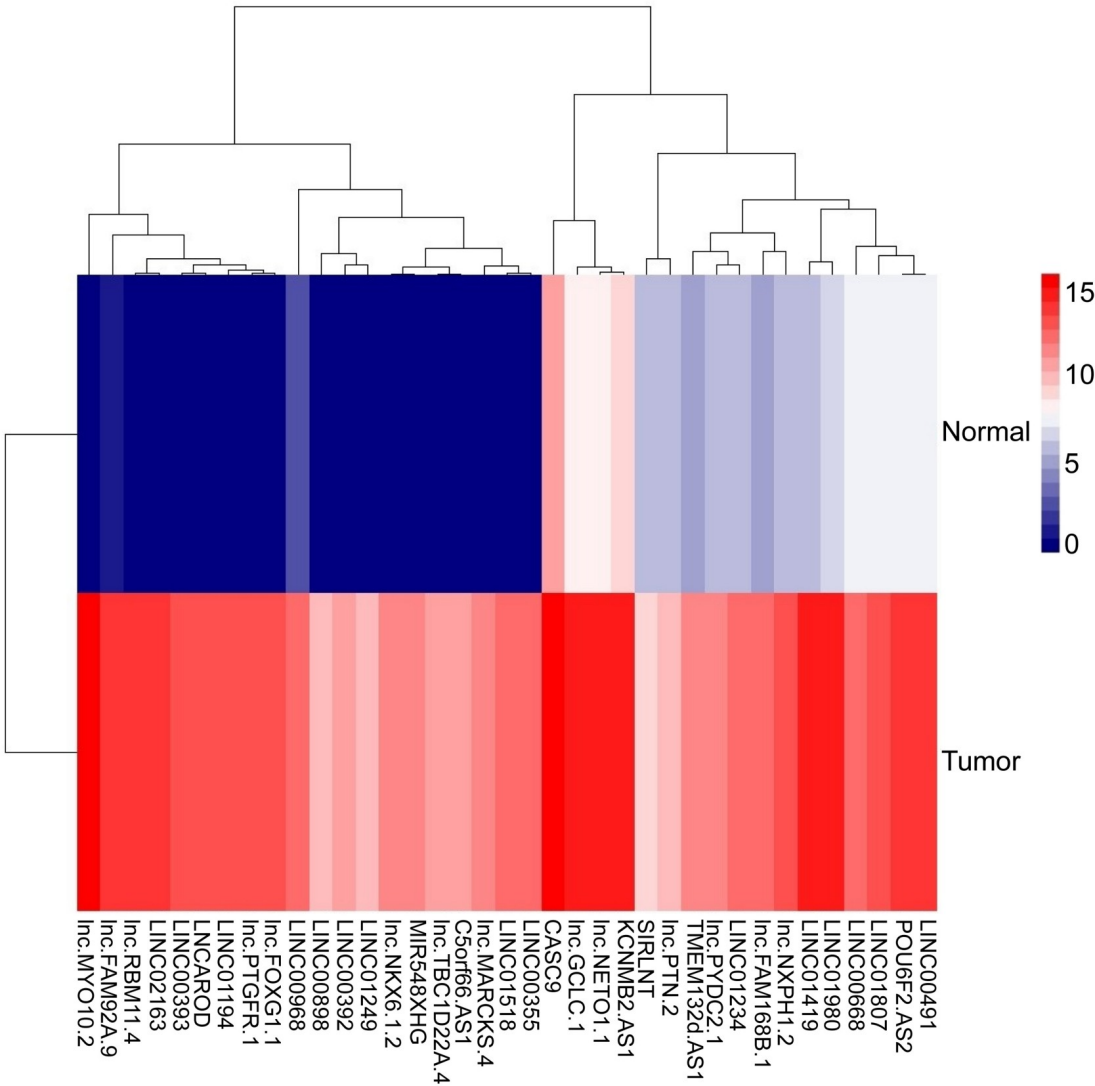
Cluster heat map of differentially expressed lncRNA in cancer tissues and adjacent tissues. The abscissa represented the differential lncRNA, and the ordinate represented the sample. The color of the heat map corresponded to the expression of lncRNA. Red indicated high expression, blue indicated low expression, p<0.05.

**Fig 3:**
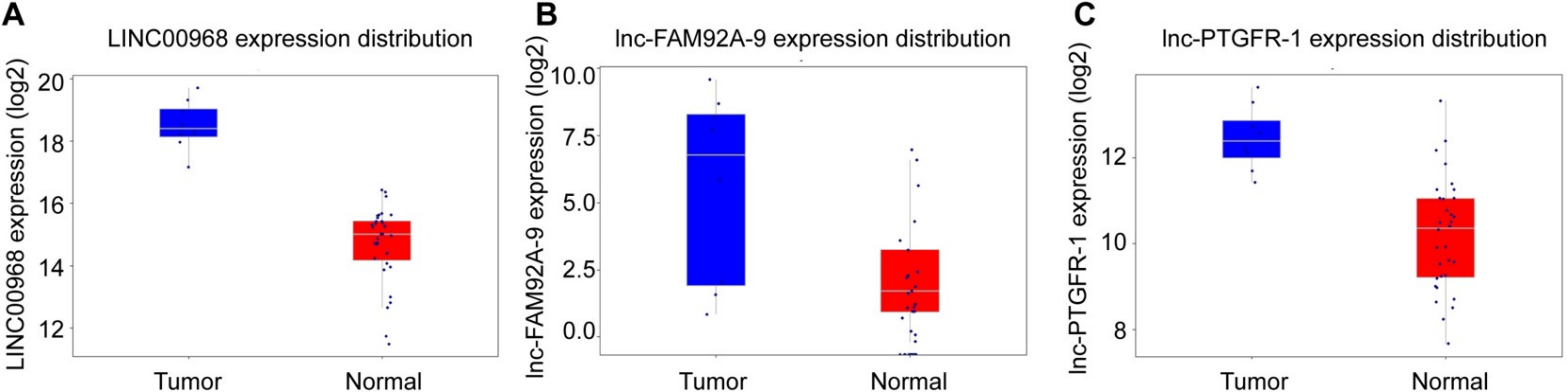
Box plot of lncRNAs differentially expressed in NSCLC tissues and adjacent tissues. A. The expression of LINC00968 in NSCLC tissues and adjacent tissues. B. The expression of lnc-FAM92A-9 in NSCLC tissues and adjacent tissues. C. The expression of lnc-PTGFR-1 in NSCLC tissues and adjacent tissues. The abscissa represented the group and the ordinate represented the expression of lncRNA, p<0.05.

Due to the poor prognosis of NSCLC, it is urgent to find a new treatment method. We used bioinformatics software to search the differentially expressed genes in the adjacent tissues and NSCLC tissue samples. In the TCGA database, we screened out relevant lncRNA genes with P<0.05 to create a volcano plot (Fig 1). The significant lncRNA (P<0.05) were selected to produce a heat map (Fig 2). The heat map showed 37 lncRNAs with significant differences between the NSCLC group and the adjacent group (P<0.05). Comparing with the adjacent group, they were all highly expressed in the NSCLC group. Three lncRNAs with significant differences of NSCLC were selected from 37 lncRNAs. LINC00968, lnc-FAM92A-9, and lnc-PTGFR-1 were utilized to make box plots (Fig 3). Compared with the adjacent group, LINC00968, lnc-FAM92A-9, and lnc-PTGFR-1 had high expression in cancer tissues. Therefore, LINC00968, lnc-FAM92A-9, and lnc-PTGFR-1 maybe target genes for regulating NSCLC.

### 3.2. Survival analysis of lncRNA in NSCLC tissues and adjacent tissues

**Fig 4:**
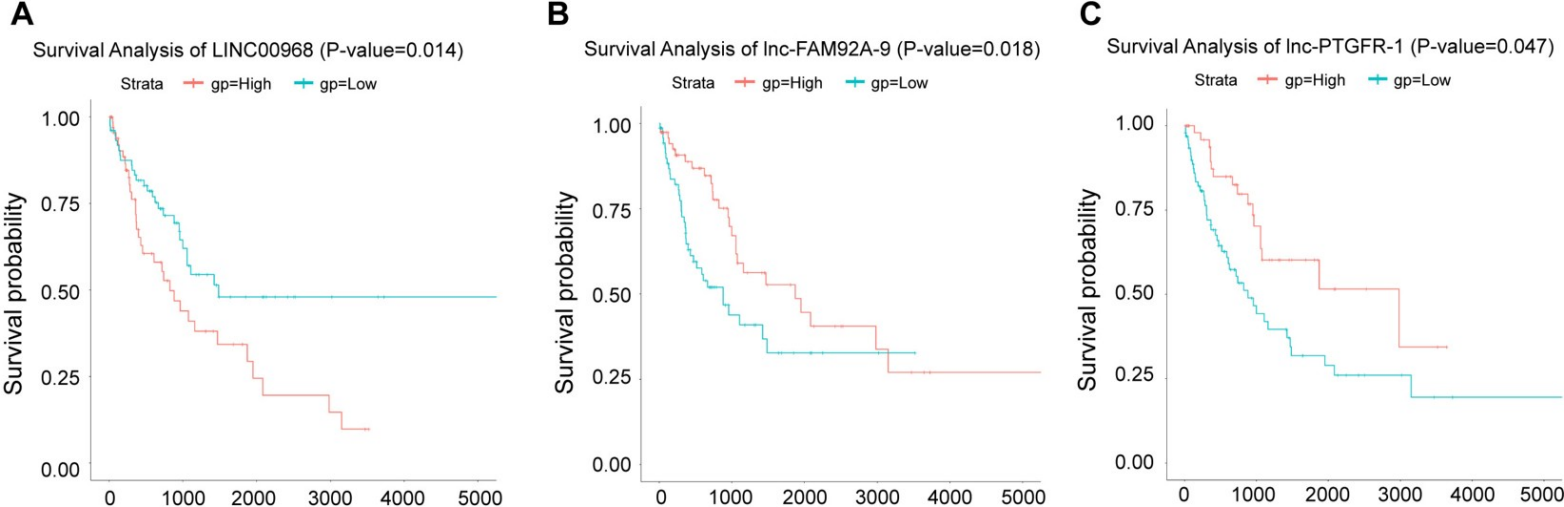
Survival curve of differentially expressed lncRNA. A. The survival curve of LINC00968. B. Survival curve of lnc-FAM92A-9. C. Survival curve of lnc-PTGFR-1. The abscissa represented survival time (days), and the ordinate represented survival rate. The blue line represented the low risk curve and the red line represented the high risk curve, p<0.05.

The Kaplan-Meier survival curves showed the correlation between disease and genes. Different gene expression affects the survival of patients. To determine the lncRNA associated with the survival of NSCLC patients, we selected three lncRNAs (LINC00968, lnc-FAM92A-9, and lnc-PTGFR-1) to make survival curves (Fig 3). Kaplan-Meier survival curves were utilized to assess the relationship between these lncRNAs and patient survival rate (Fig 4). According to our results, patients with high expression of LINC00968 had a much shorter survival time. Patients with high expression of lnc-FAM92A-9 and lnc-PTGFR-1 had a much longer survival time. Thus, LINC00968 may be a biomarker for NSCLC.

### 3.3. Analysis of mRNA expression and survival in NSCLC tissues and adjacent tissues

**Fig 5:**
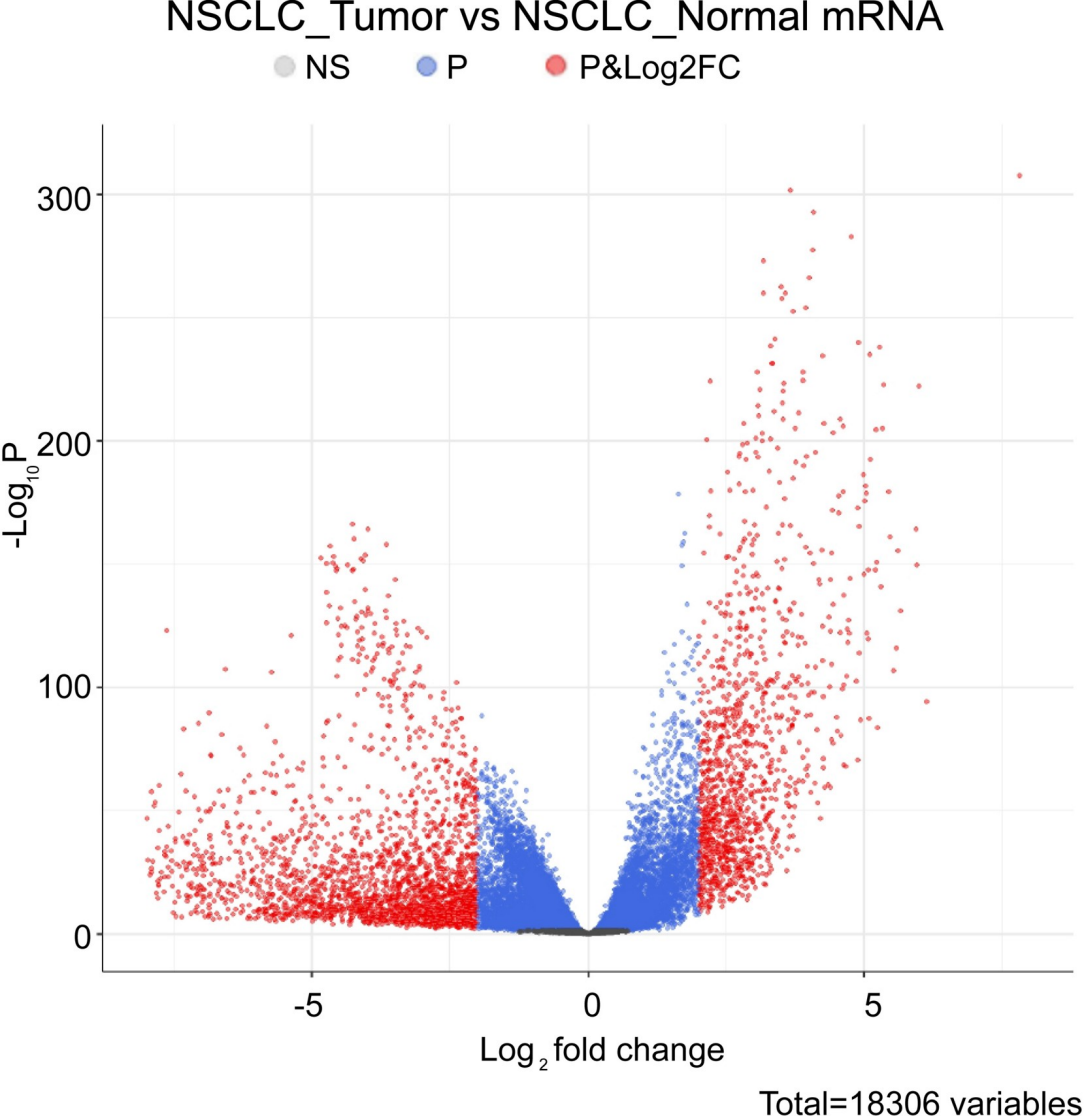
Differential expression of mRNA volcano in cancer tissues and adjacent tissues. The ordinate was p-value. The log2 value of the screening condition was used as the abscissa, p<0.05. Red represents genes with fold change (FC) > 2 and P < 0.05.

**Fig 6:**
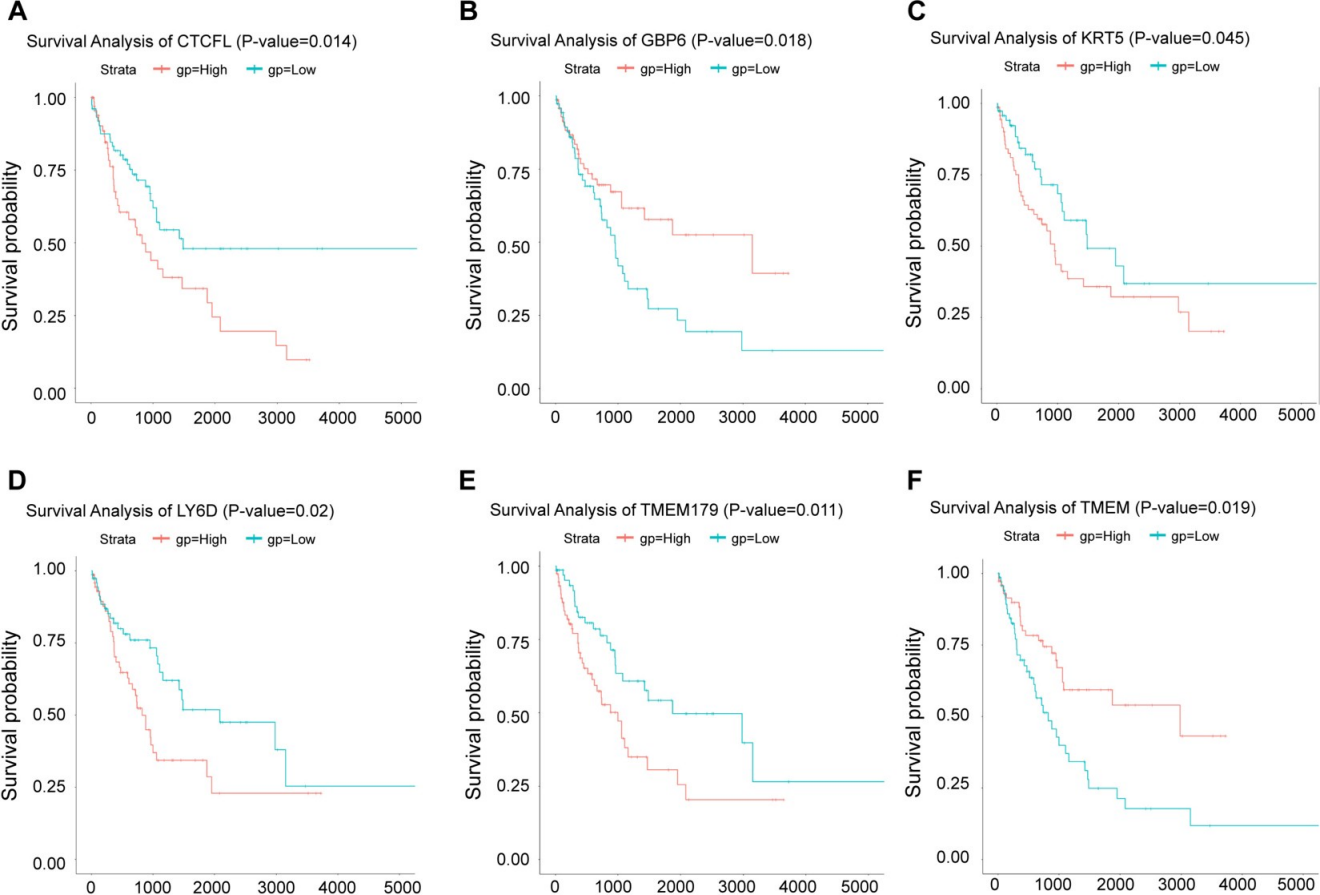
Survival curve of differentially expressed mRNA. A. Survival curve of CTCFL. B. Survival curve of GBP6. C. Survival curve of KRT5. D. Survival curve of LY6D. E. Survival curve of TMEM. F. Survival curve of TMEM179. The abscissa represented survival time (days), and the ordinate represented survival rate. The blue line represented the low risk curve and the red line represents the high risk curve, p<0.05.

To screen out the differentially expressed mRNAs in the adjacent tissues and NSCLC tissue samples, we screened and drew a volcano plot of mRNA (Fig 5). Highly correlated mRNAs were selected from volcano plots and made Kaplan-Meier curves. We found that 6 mRNA expressions were significantly different (P<0.05), including GBP6 (guanylate binding protein), TMEM (transmembrane protein), CTCFL (liver cancer marker gene), KRT5 (keratin 5), LY6D (lymphocyte antigen) and TMEM179 (transmembrane protein). In mRNA prognostic survival curve analysis (Fig 6), we found GBP6 and TMEM were positively correlated with the patient’s overall survival, with high expression and prolonged survival time. CTCFL, KRT5, LY6D, and TMEM179 were all highly expressed in NSCLC patients, but the survival rate of patients was low. Therefore, CTCFL, KRT5, LY6D, TMEM179 may be related to the prognosis of NSCLC.

### 3.4. The functional classification of mRNA was annotated by GO term and KEGG pathway

**Fig 7:**
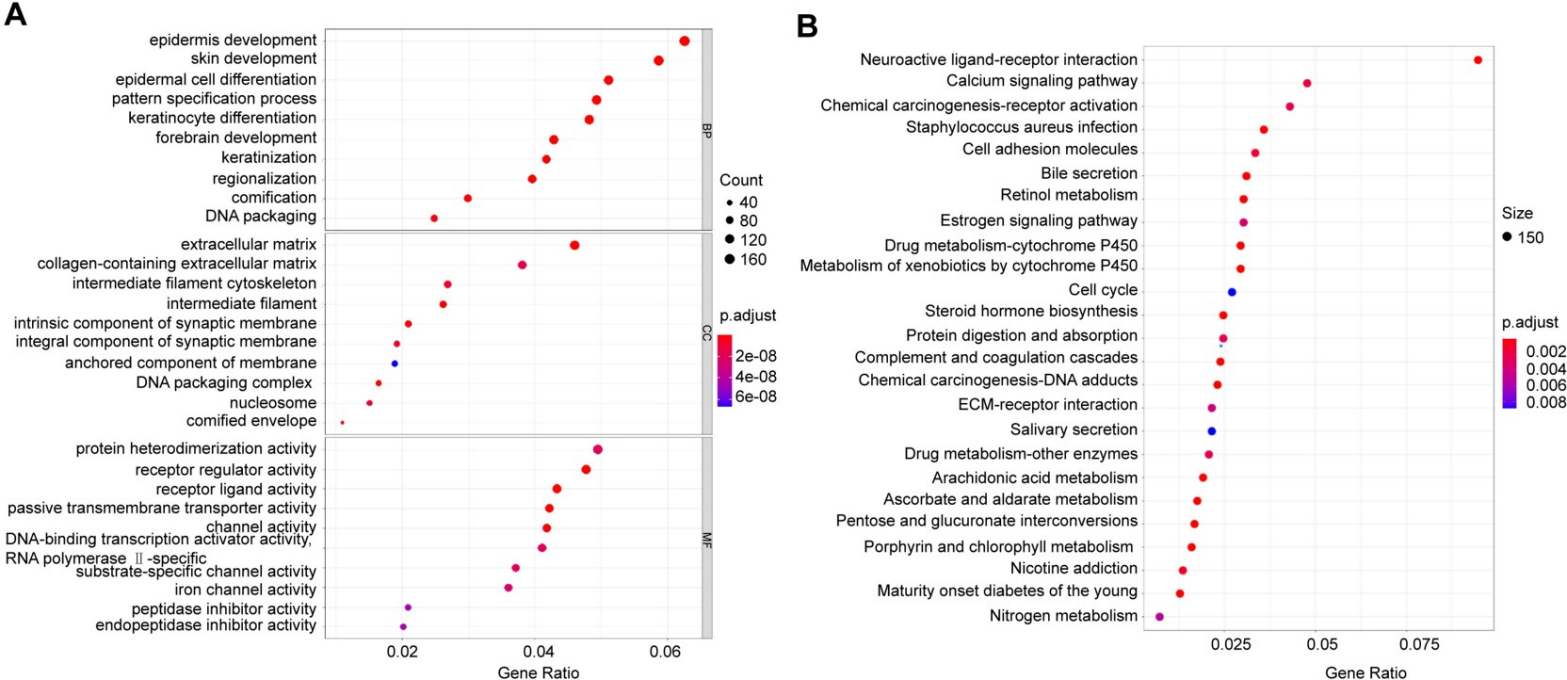
GO analyze and KEGG pathway. A. BP: biochemical process, CC: cell component, MF: molecular function, the abscissa is enriched to this term. The number of different genes, p<0.05.

The GO term simply annotates gene products from the function, biological pathways involved, and location in the cell. To better explore the function of mRNA, the clusterProfiler package was utilized to make a GO term of these mRNAs (Fig 7). In the biochemical process (BP), the functions of these mRNAs are predominantly concentrated in regionalization, cornification and DNA packaging. In the cell component (CC), the functions of these mRNAs were concentrated in components such as the intermediate filament and the intrinsic component of the synaptic membrane. In molecular function (MF), the functions of these mRNAs focus on channel activity (channel activity) and receptor regular activity (regulatory receptor activity). However, in the biochemical process, the epidermal development function, endopeptidase inhibitor activity in the molecular function, the cornified envelope (encoding keratinized envelope) function in the cell components are in a low expression state.

We also performed KEGG enrichment analysis and found several pathways significantly related to NSCLC. These pathways including Neuroactive ligand-receptor interaction pathway, Calcium signaling pathway, Chemical carcinogenesis-receptor activation pathway, Staphylococcus aureus infection pathway, Cell adhesion molecules pathway, Bile secretion pathway, Retinol metabolism pathway, Estrogen signaling pathway, Drug metabolism-cytochrome P450 pathway, Metabolism of xenobiotics by cytochrome P450 pathway, Cell cycle pathway, et al.

### 3.5. GESA in GO term and KEGG pathway

**Fig 8:**
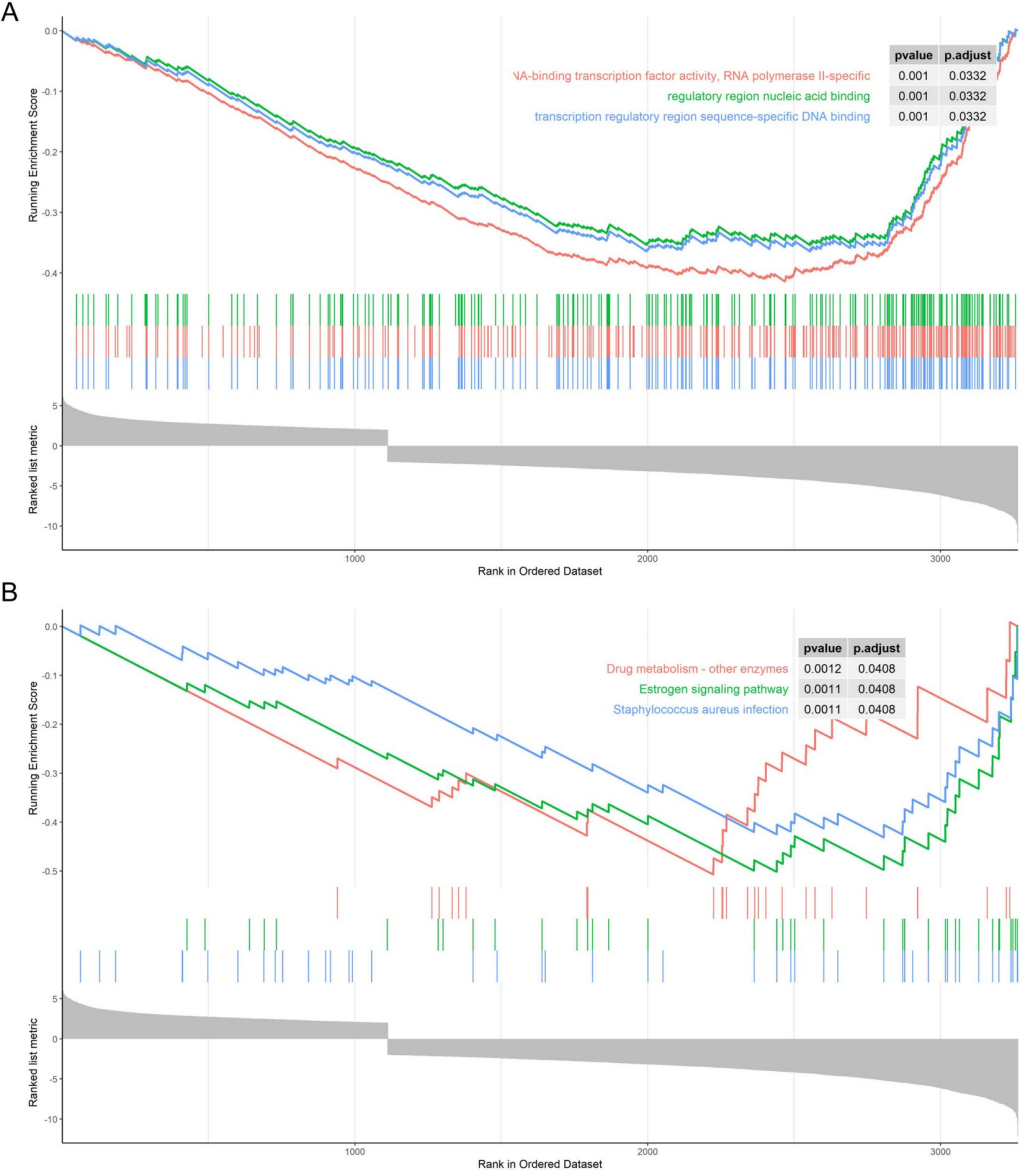
GESA in GO term and KEGG pathway. A. GSEA in GO term. B. GSEA in KEGG pathway.

At the cellular level, GSEA revealed that NSCLC was significantly related to RNA-binding transcription factor activity, RNA polymerase II-specific, nucleic acid binding effect in the regulatory region, and the binding between transcription regulatory region sequence and DNA (Fig 8A). At the metabolic level, GSEA revealed that NSCLC was enriched in the Drug metabolism-other enzymes pathway, Estrogen signaling pathway, and Staphylococcus aureus infection pathway (Fig 8B).

### 3.6. Construction of ceRNA network of lncRNA and mRNA in NSCLC tissues and adjacent tissues

**Fig 9:**
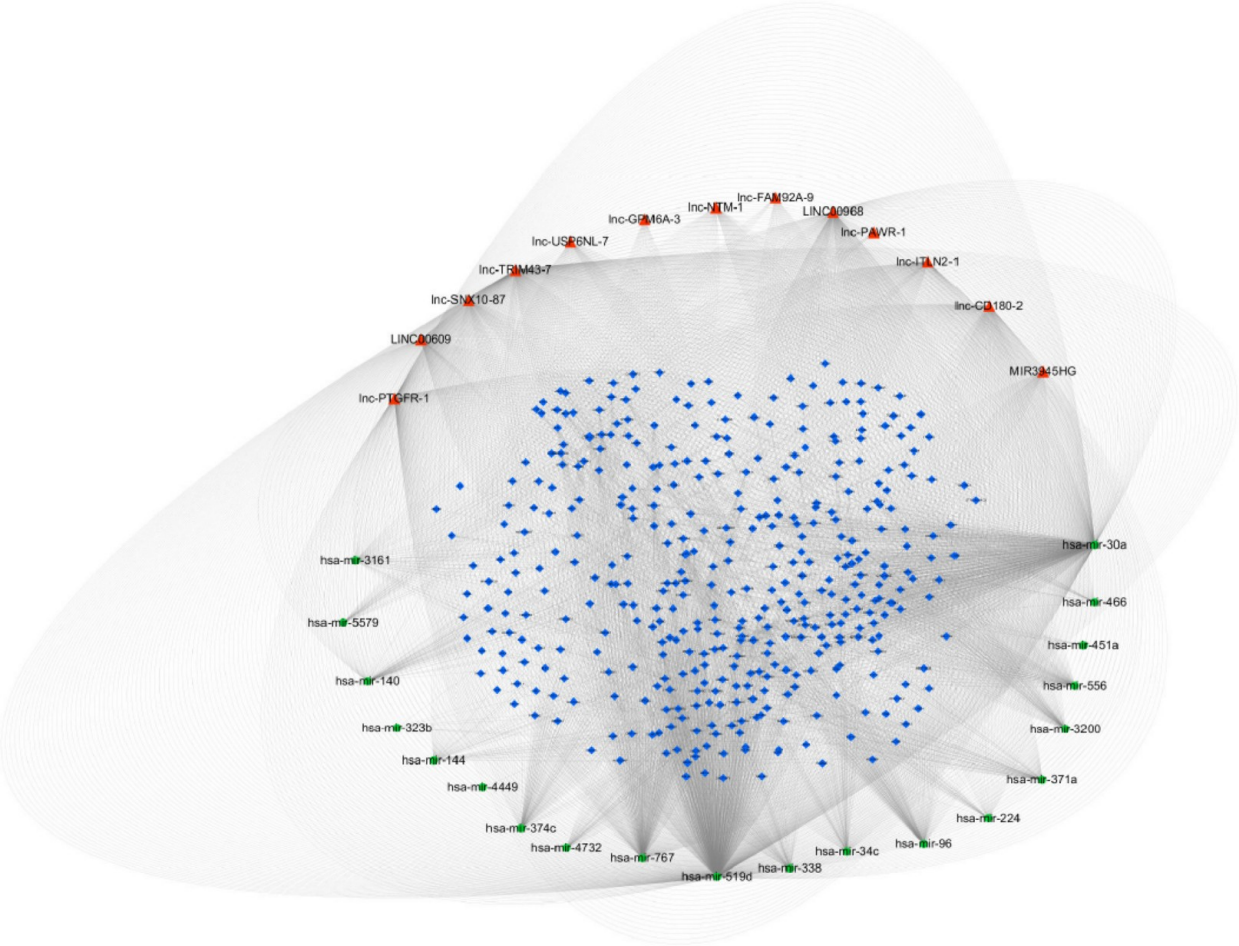
CeRNA ternary diagram of lncRNA-miRNA-mRNA. The red triangle represented lncRNA, the green dot represented miRNA, and the blue diamond represented mRNA. Significantly differentially expressed miRNA and lncRNA regulatory network, p<0.05.

LncRNA could affect the abundance of target gene mRNA and protein level by adsorbing miRNA. Based on the 5 lncRNAs analyzed in the survival curve, we took the lncBase and miRTarBase databases to predict other lncRNAs that interact with these 5 lncRNAs to construct a lncRNA-miRNA-mRNA interaction network (Fig 9). A total of 13 lncRNAs (red triangles) and 20 miRNAs (green dots) interacted with each other in this network. At the same time, lncRNA LINC00968, lnc-FAM92A-9 and lnc-PTGFR-1 also interacted with related miRNAs in the ceRNA network. By topological analysis, the expression of hsa-miR-519d and hsa-miR-30a in miRNAs in the ceRNA network were more significant (P<0.05).

### 3.7. Verification of differentially expressed lncRNA in BEAS2B and A549

**Fig 10:**
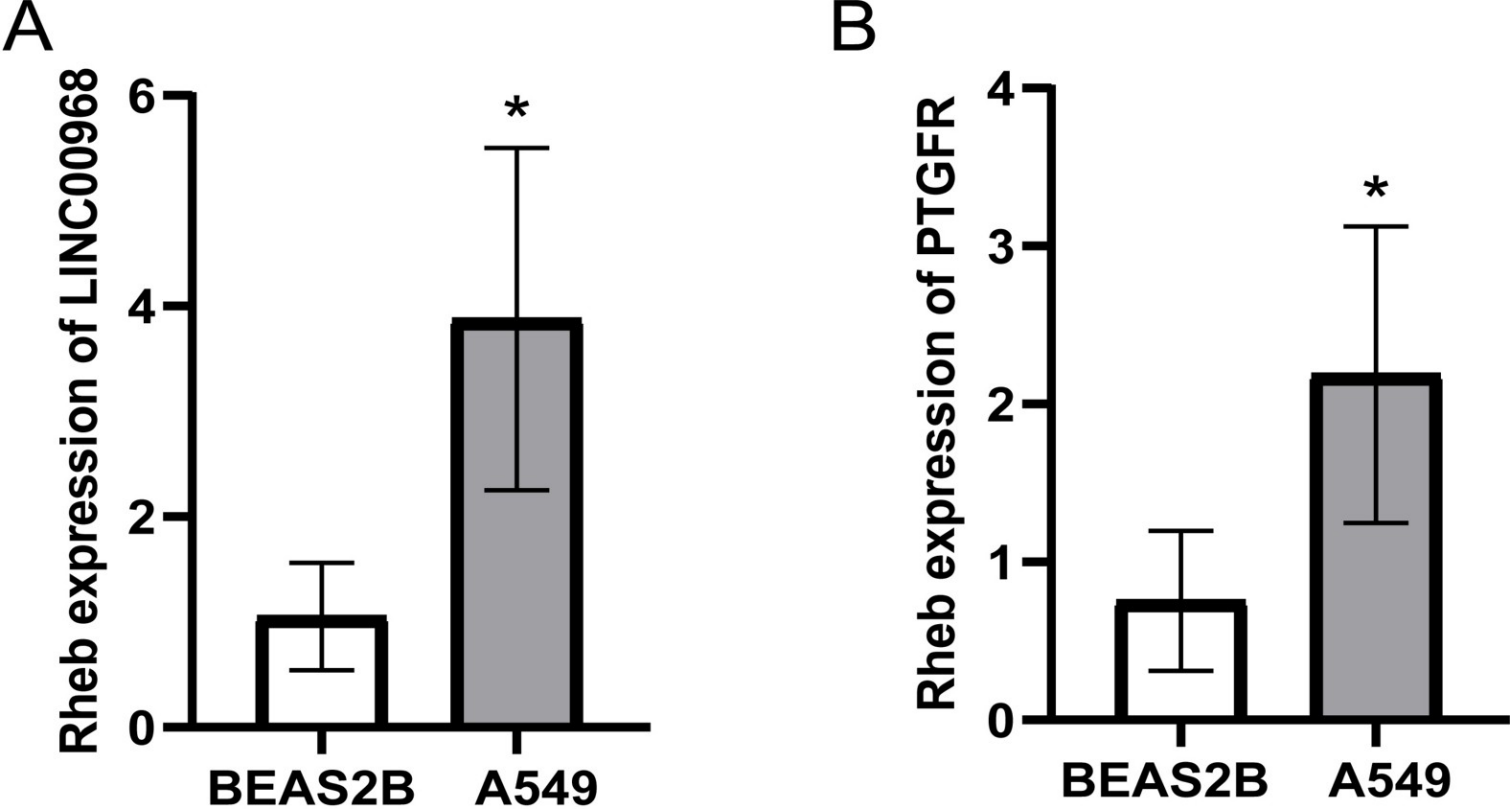
Verification of differentially expressed mRNA in BEAS2B and A549. LINC00968 (A) and PTGFR (B) were detected by qRT-PCR.

The preliminary bioinformatics prediction results indicated that LINC00968, lnc-FAM92A-9 and lnc-PTGFR-1 might be the target genes for regulating NSCLC. The expression of lnc00968 and PTGFR was determined by qRT-PCR in BEAS2B and A549 cells. The results showed that the expression of lnc00968 and PTGFR in A549 was significantly higher than that of BEAS2B (Fig 10). This is consistent with the results predicted by our bioinformatics. The LINC00968 and lnc-PTGFR-1 in NSCLC tissues were higher than that in adjacent tissues. It showed that LINC00968 and lnc-PTGFR-1 could indeed promote the occurrence of NSCLC.

### 3.8. Verification of lncRNA differentially expressed in BEAS2B and A549

**Fig 11:**
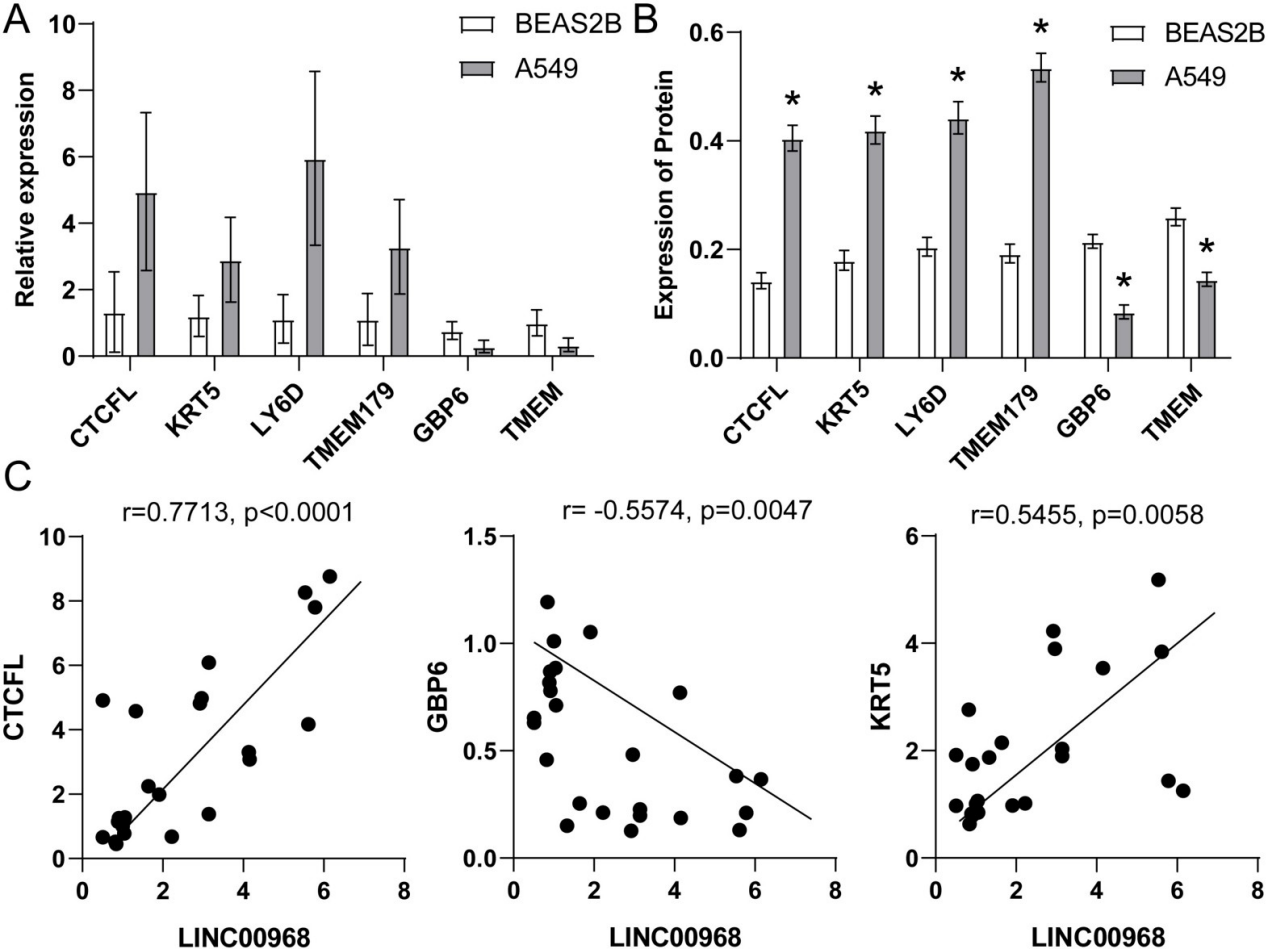
Verification of mRNA differentially expressed in BEAS2B and A549. qRT-PCR (A) and WB (B) were used to detect the expression levels of CTCFL, KRT5, LY6D, TMEM, GBP6, TMEM179. (C) Pearson analysis was used to detect the correlation between LINC00968 and CTCFL, GBP6, KRT5.

Our bioinformatics prediction results showed differentially expressed mRNAs in NSCLC and adjacent tissues, including CTCFL, KRT5, LY6D, TMEM179, GBP6, TMEM, etc. qRT-PCR and WB were performed to reveal the expression of these mRNAs in BEAS2B and A549 cells. The results unveiled that the expression of CTCFL, KRT5, LY6D, and TMEM179 in A549 was higher than that in BEAS2B. And the expression of GBP6 and TMEM in A549 is lower than that of BEAS2B (Fig 11A and 11B). We analyzed the correlation between the expression of CTCFL, GBP6, KRT5, and LINC00968 (Fig 11C). The results indicated that CTCFL and KRT5 were positively correlated with the LINC00968 expression, and GBP6 was negatively correlated with LINC00968 expression. It showed that CTCFL, GBP6, and KRT5 were indeed associated with LINC00968.

### 3.9. LINC00968 regulated A549 cell proliferation and apoptosis

**Fig 12:**
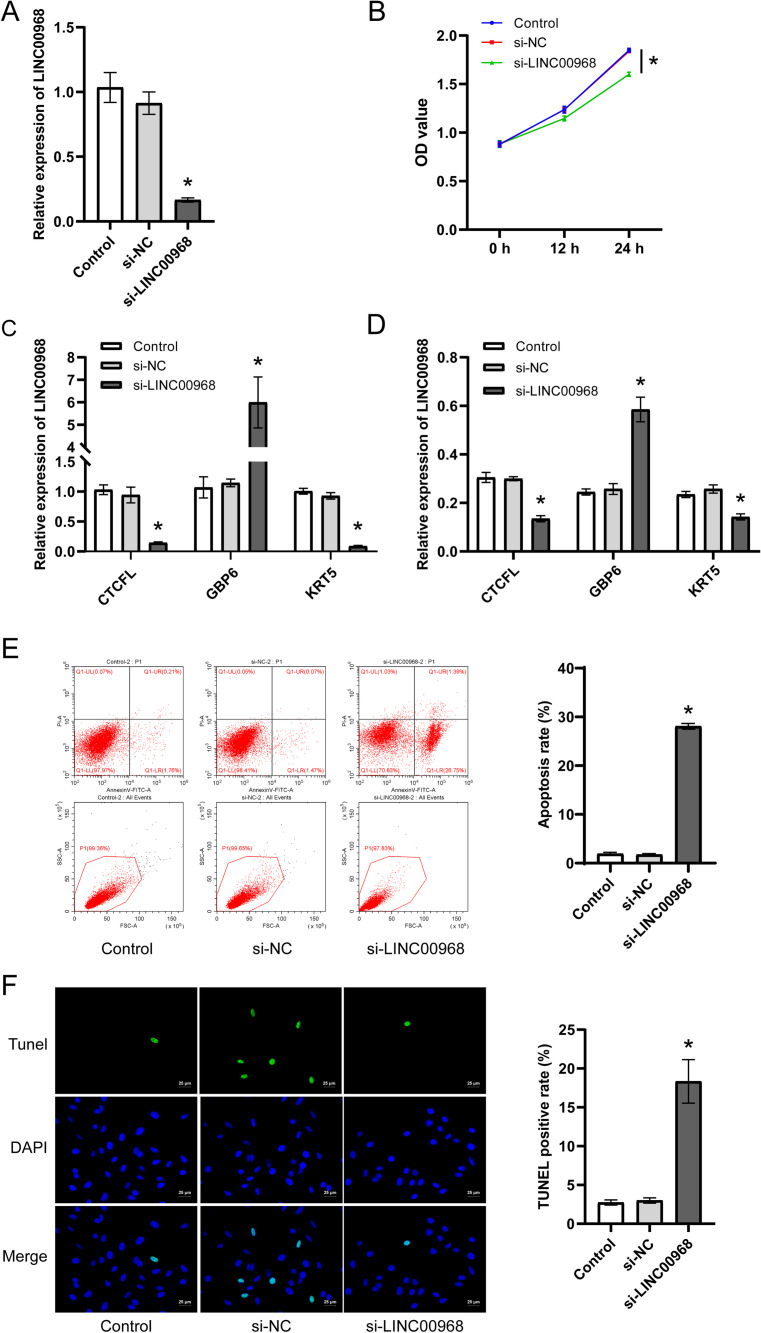
LINC00968 regulated A549 cell proliferation and apoptosis. (A) qRT-PCR was explored to examine whether knockdown of LINC00968 is successful. (B) CCK8 was performed to measure cell proliferation. qRT-PCR (C) and WB (D) were used to detect the expression of CTCFL, GBP6, KRT5. (E) Flow cytometry was utilized to detect cell apoptosis. (F) Tunel was explored to determined cell apoptosis.

**Fig 13:**
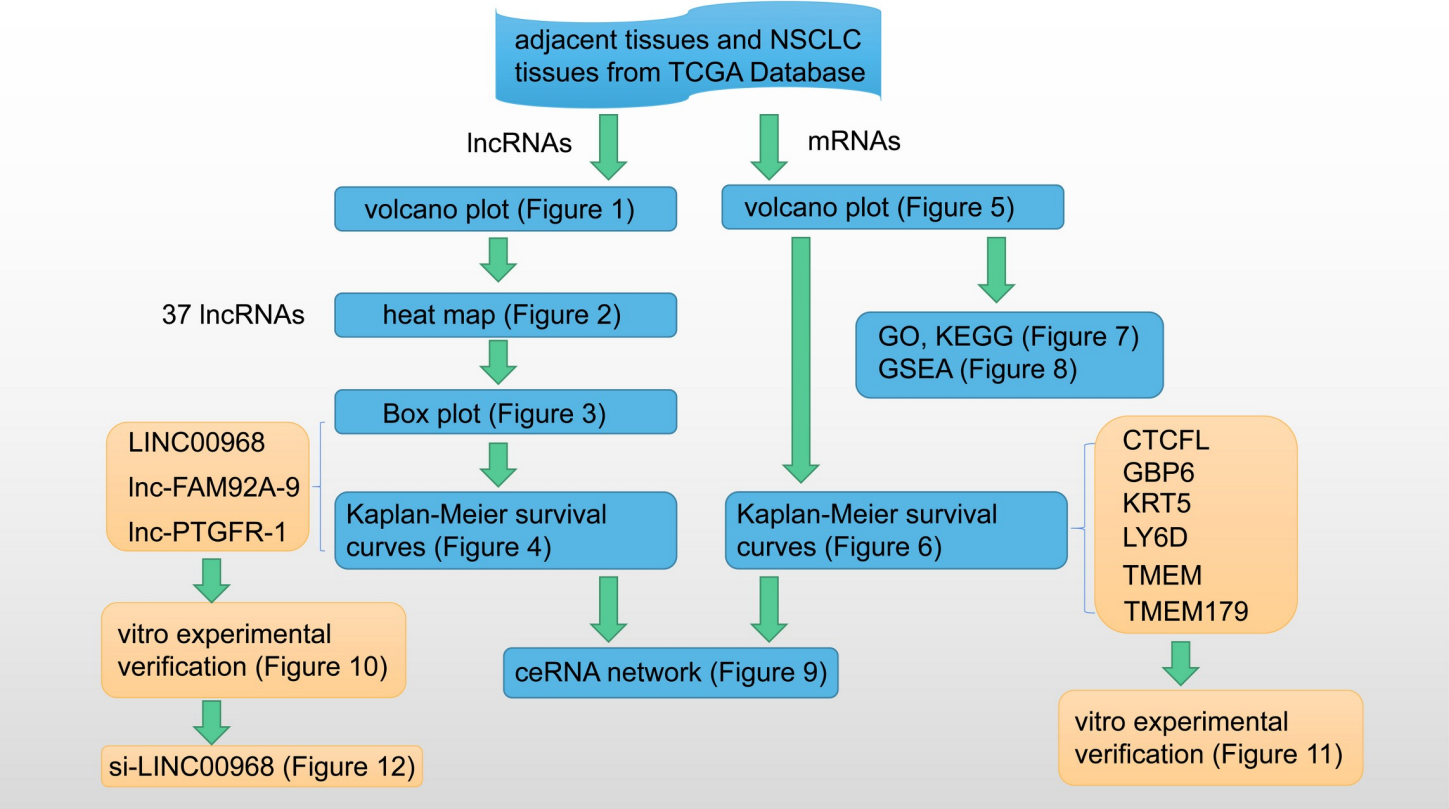
Research process display.

In order to verify whether LINC00968 regulates NSCLC, we used si-LINC00968 to knock down the expression of LINC00968 in A549 cells (Fig 12A). CCK8 was used to confirm cell proliferation. After the cells were treated for 24 h, compared with the Control group, the proliferation of A549 cells in the si-LINC00968 group was significantly inhibited (Fig 12B). Then, we used qRT-PCR and WB to determine the expression of CTCFL, GBP6, and KRT5 in each group. We found that knocking down LINC00968 significantly inhibited the expression of CTCFL and KRT5, and promoted the expression of GBP6 (Fig 12C and 12D). It further showed that LINC00968 regulated CTCFL, GBP6, and KRT5. Finally, we used flow cytometry and Tunel assay to detect cell apoptosis. And we found that the apoptosis of cells in the si-LINC00968 group was significantly increased (Fig 12E and 12F). All the processes of this study were shown in Fig 13.

## 4 Discussion

NSCLC accounted for 80% of lung cancers and rapidly spread and high mortality [29]. The existing treatment methods for NSCLC were mainly traditional monoclonal antibody targeted therapy and radiotherapy and chemotherapy [30]. At present, basic research on lncRNA targeted therapy has been carried out on a variety of cancers. LncRNA has a characteristic expression pattern in tumors. Peng et al. found knocking down the expression of the lncRNA (HOXA-AS2) gene in gallbladder cancer cells suspended the G1 phase of the cell cycle and inhibited the proliferation *in vitro* experiments [31]. LncRNA GClnc1 promoted the proliferation and invasion of bladder cancer tissues by activating MYC proto-oncogene [32]. In NSCLC, some researchers proved that LncRNA TUC338 promoted cancer invasion by regulating the MAPK pathway [33]. However, current bio-information analysis and basic research at the genetic level of NSCLC are still limited.

In this study, we analyzed the sequences of lncRNA and mRNA in NSCLC tissues and adjacent tissues. Compared with adjacent tissues, our results showed significant differences in the expression of lncRNA sequences and mRNA sequences in cancer tissues. In accordance with the differentially expressed lncRNAs, a volcano plot and heat map were drawn. And box plots were shown for the lncRNAs that were significantly differentially expressed. Finally, we found that the expression of LINC00968, lnc-FAM92A-9 and lnc-PTGFR-1 in cancer tissues was lower than in normal tissues. We made a survival curve analysis on the prognosis of these lncRNAs. It was found that when lnc-FAM92A-9 and lnc-PTGFR-1 were highly expressed, the survival level of patients was positively correlated. However, the late data of patients with high expression of lnc-PTGFR-1 and lnc-PTGFR-1 was censored, the mortality rate might have been higher, but the data might not be fully recorded. The expression of LINC00968 was negatively correlated with the patient’s survival. Combined with the survival analysis results, we detected the expression of LINC00968 and PTGFR in BEAS2B and A549. These results were consistent with the bioinformatics results. Both LINC00968 and lnc-PTGFR-1 were highly expressed in A549. Thus, LINC00968 and lnc-PTGFR-1 could positively regulate NSCLC. Gang Liu et al. found that reducing the expression of LINC00968 *in vitro* experiments reduced the growth and proliferation of osteosarcoma cells through the PI3K/AKT/mTOR pathway [34]. However, there are still few studies involved with lnc-PTGFR-1 and lnc-FAM92A-9, so our future interests for research could focus on these two lncRNAs.

Clinically, testing the clinical survival time of patients was a long-term experiment. We could simply infer the impact of a certain gene on the patient’s survival time through survival analysis [35]. We created a volcano plot in accordance with mRNAs with different expressions, and selected significantly different mRNAs for prognostic survival curve analysis. We observed these mRNAs have a significant impact on the survival of patients in the survival curve. GBP6 and TMEM were positively correlated with overall survival. CTCFL was negatively associated with the overall survival of patients. We tested the expression of CTCFL, KRT5, LY6D, TMEM179, GBP6, and TMEM in BEAS2B and A549. Then, we found that CTCFL, KRT5, LY6D, and TMEM179 in A549 were higher than that in BEAS2B, while GBP6 and TMEM were just the opposite. The results indicated that these 6 mRNAs might be able to regulate NSCLC. Besides, CTCFL, LY6D, TMEM179, and KRT5 may be related to confirmed p53, INK/ARF and other oncogenes disorders [36]. NSCLC has a high fatality rate due to its metastasis and poor prognosis. LY6D has been proved to be an effective marker involved in the metastasis of estrogen receptor-positive breast cancer [37]. It is speculated that regulating LY6D reduced the probability of NSCLC metastasis [38]. GBP6 has been confirmed to have a poor prognosis in tongue squamous cell carcinoma (TSCC) patients due to its low expression in TSCC. Therefore, we speculated that regulating GBP6 was an effective way to improve the poor prognosis of NSCLC [39]. To further determine the regulatory effect of LINC00968 on NSCLC, LINC00968 in A549 cells was knocked down. The results indicated that LINC00968 promoted cell apoptosis and inhibited cell proliferation. and control CTCFL, GBP6, and KRT5.

It is convenient and effective to apply GO and KEGG functional enrichment analysis and perform GSEA analysis to find therapeutic candidates for the disease [40]. From GO term, We found that both the up- and down-regulated mRNAs indicated regionalization, keratinization, and DNA packaging in the biochemical components; intermediate filament and synaptic membrane in cell composition; channel activity and receptor activity in molecular function. We speculated that these mRNAs regulated the proliferation and differentiation of cancer cells by participating in cell cycle activities. In the KEGG pathway analysis, we got possible key pathways in NSCLC was regulated by Neuroactive ligand-receptor interaction pathway, Calcium signaling pathway, Chemical carcinogenesis-receptor activation, Staphylococcus aureus infection, Cell adhesion molecules, et al. GSEA further revealed that NSCLC was related to RNA-binding transcription factor activity, RNA polymerase II-specific, regulatory region nucleic acid binding, transcription regulatory region sequence-specific DNA binding, drug metabolism-other enzymes, Estrogen signaling pathway, and Staphylococcus aureus infection.

The construction of the ceRNA network helped us to find out effective targets for the treatment of NSCLC. Kong et al. sequenced A549 and HCC827 cells and constructed 33 lncRNA-miRNA-mRNA pathways in the drug-resistant NSCLC ceRNA network. In this network, 12 dysregulated lncRNAs, 5 dysregulated miRNAs, and 8 dysregulated mRNAs were found. These RNAs may be potential biomarkers for the treatment of NSCLC. It provided a basis for further animal experiments and clinical experiments [22]. The study of lncRNA-miRNA-mRNA interaction required a lot of experimental support [41–43]. Therefore, it was very necessary for the network to predict the target [44–47]. We conducted a comprehensive analysis of lncRNA, miRNA, and mRNA sequences and obtained the regulatory ceRNA network. 13 lncRNAs were interacting with 20 miRNAs in the ceRNA network. In the ceRNA network, the expression of two miRNAs, hsa-miR-519d, and hsa-miR-30a were more significant. Hsa-miR-519d was considered to be involved in signal transduction, cell growth and death and other metabolic processes. And it is speculated that hsa-miR-519d probably became a target for regulating gastric cancer progression [48]. The difficulty in the prognosis of NSCLC was due to its drug resistance. Studies had confirmed that NSCLC had developed resistance to various chemotherapeutic drugs such as cisplatin [49]. Hu S et al. indicated that hsa-miR-30a was related to and resistant to NSCLC, which could be a potential treatment for NSCLC resistance [50]. We admitted that a small number of samples might cause relatively large noise. However, we used Gpower to calculate the validity of the sample number, which was higher than 80%. Therefore, the sample size was still meaningful for this study. In addition, we finally verified the results by cytology, and obtained satisfactory results. Due to the limited time and funds, we have not added clinical sample analysis to this project. However, we also believe that the analysis results of clinical samples have a good reference value for this study. Therefore, we will study the correlation between mRNA expression and the clinical stage in the future.

## 5 Conclusion

Our study compared the sequences of NSCLC tissues and normal adjacent tissues in the TCGA database. As a result, we got 3 significantly differently expressed lncRNAs of LINC00968, lnc-FAM92A-9 and lnc-PTGFR-1 by bioinformatics analysis. We also found 6 mRNAs (CTCFL, KRT5, LY6D, TMEM179, GBP6, and TMEM) significantly impacted patient survival. These mRNAs and lncRNAs were involved in the same ceRNA network. In the future, we will conduct clinic experiments based on these genetic data and hope to find new treatments for NSCLC for clinical treatment.
